# Ten simple rules for getting and giving credit for data

**DOI:** 10.1371/journal.pcbi.1010476

**Published:** 2022-09-29

**Authors:** Elisha M. Wood-Charlson, Zachary Crockett, Chris Erdmann, Adam P. Arkin, Carly B. Robinson

**Affiliations:** 1 Environmental Genomics and Systems Biology Division, E.O. Lawrence Berkeley National Laboratory, Berkeley, California, United States of America; 2 Biosciences Division, Oak Ridge National Laboratory, Oak Ridge, Tennessee, United States of America; 3 American Geophysical Union, Washington, DC, United States of America; 4 U.S. Department of Energy Office of Scientific and Technical Information, Oak Ridge, Tennessee, United States of America; Carnegie Mellon University, UNITED STATES

## Introduction

Data is a hot topic and for good reason—it can be challenging to generate, complicated to analyze, difficult to describe, and near impossible to share. But, unlike the scientific publication, data by itself gets almost no credit or recognition beyond a few figures in the paper. However, keeping data hidden until publication delays knowledge transfer and slows down discovery. In addition, the criteria for publication continues to grow more demanding, as does the need to find new ways to share information [1]. Imagine if science was able to effectively and efficiently share data in a findable, accessible, interoperable, and reusable (FAIR; [2]) manner outside of a publication, in a way that enables comparability and reproducibility, while also ensuring that the data contributors were appropriately credited for their contributions! Could we worry less about being scooped? Should we embrace data sharing as a desirable criteria, in addition to publishing, during career evaluations [3]? Can we accelerate the process of science?!

Good news is some of this is already happening. The rise of preprints has demonstrated that sharing science prior to publication can respect one’s individual contribution while also accelerating scientific knowledge transfer [4,5]. For example, rapid dissemination of knowledge via preprints was critical in supporting a rapid response to the recent global pandemic [6,7]. If you are interested in submitting a preprint but don’t know where to start, consider the 10 Simple Rules article by Bourne and colleagues [8]. In addition to preprints, many publishers support data papers (Nature, Elsevier, Springer, etc.), some research domains have started to advocate for data sharing (e.g., biodiversity; [9–11]), and a few data centers even facilitate data sharing by offering data preprints (e.g., NASA’s Distributed Active Archive Centers). In addition, federal agencies, including funders like NSF and NIH, data producers like NASA, and a coalition of scientific societies, publishers, institutions, etc. are beginning to advocate for the *practice of referencing or citing data* [12–15].

This article attempts to summarize current best practices that support the movement towards enabling researchers to cite and receive credit for their data. The authors are a small representation of the people and organizations trying to make this happen, and we acknowledge that it is not possible to capture all efforts behind this endeavor in 10 Simple Rules. We encourage interested readers to dive deeper by providing related resources along the way.

### Rule 1: Appreciate the FAIR data principles for what they are (and are not)

Any article that talks about giving credit for data must support the FAIR Data Principles [2]. By ensuring that data are FAIR, authors can immediately make data more discoverable and easier to reuse. FAIR also emphasizes that the information describing the data should be machine-readable, enabling automated attribution. Several resources provide details and criteria for evaluating FAIR (e.g., [16]), so we won’t belabor those points here. We do, however, want to note some common misinterpretations around FAIR.

#### Findable

The first pillar of FAIR is Findable—if you can’t find data, the rest is meaningless. The data should be **uniquely identified with a persistent identifier** (PID). A common data PID is a digital object identifier (DOI). PIDs are universal, globally unique, and machine readable, which allows resources to be referenced by a single identifier. That identifier can remain constant even if the URL for the resource is changed. Also, PIDs should be registered and indexable, so they are discovered by search engines. Many resources, such as DataCite [17], are tracking data DOIs. *Note*: *If you might need to update reference information or source*, *be sure the PID can be versioned*.

#### Accessible

The second pillar of FAIR is accessible, which enables data to be accessed once found. This requires data to be **retrievable with a standard protocol**, such as HTTPS, ensuring that others can make use of the data. However, accessible does not necessarily equate to open, nor should it. There are situations where data should only be used under certain conditions and with permission. For instance, clinical data may require additional criteria for access so that patient privacy is protected. Instead, accessibility requires at least that the conditions by which data can be obtained are public. *Note*: *Data access does not mean fully open and may be restricted to comply with research and clinical ethics*. *See Rules 1 and 2 by Contaxis* and colleagues [18].

#### Interoperable

The third pillar of FAIR is interoperable. Data should be stored in **file formats that are readily understood and widely used**. For example, common file types such as comma-separated values (CSV) are preferred over private, proprietary, or nonstandard file types. *Note*: *Interoperability of file formats does* *not* *imply data are comparable (see Rule 2)*.

#### Reusable

The final pillar of FAIR is reusable. To make data reusable, it is important to **contextualize the data with provenance and information about prior use** of the data. This makes it easier to compare with subsequent uses. *Note*: *As with interoperable*, *this does* *not* *imply data are comparable*, *able to be reproduced from scratch*, *or have been evaluated for quality unless explicitly stated (see Rule 2)*.

The principles of FAIR data can guide your data management practices and streamline downstream use and reuse of your data. They create the foundation for our culture change towards citing data.

### Rule 2: Metadata make data FAIR, comparable, and reproducible

In Rule 1, when we describe FAIR, we purposely don’t use the term “metadata,” even though it is explicitly stated throughout the formal descriptions of FAIR. This is because metadata (defined as “data that describes other data”) can mean something different to everyone, as it depends on the frame of reference defined by the “other data.” PID metadata describes high-level features of a particular data set (who created it, when it was created, etc.; see Rule 5 by Contaxis and colleages [18] for additional details), but PID metadata are not sufficient to inform about data comparability or reproducibility. At a more granular level, each research domain will have different requirements for describing the context around data collection and preparation (important for determining whether data sets can be combined or compared) and steps used for data processing (important for ensuring reproducibility). For example, different metadata are necessary when describing discrete physical samples or continuous instrument measurements, samples collected in the environment or from a lab experiment, or to describe model runs. Rule 7 by Hart and colleagues [19] has more details regarding metadata as it relates to digital data storage, and Sielemann and colleagues [20] have examples demonstrating the reuse of life science data. Finally, the US Geological Survey (USGS) has great resources on data management, including standards and metadata reporting [21].

**Fig 1:**
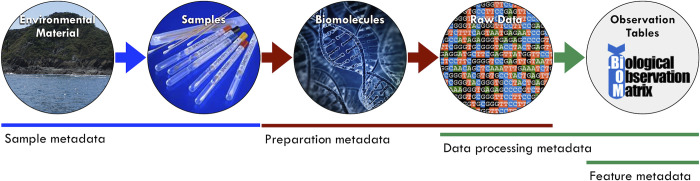
Examples of different types of metadata needed to describe the conversion of physical environmental samples into data and results. Submitting data to central repositories typically requires sample and preparation metadata. Data processing and feature metadata are generated during analysis. *Credit*: *Luke Thompson*, *PhD (National Oceanic and Atmospheric Administration)*. *Source*: *the National Microbiome Data Collaborative* [22]).

Here, we describe an example of the different layers of metadata (Fig 1 and Table 1) recommended to make data derived from physical samples collected from the environment (e.g., soil or water), processed in the laboratory (e.g., DNA sequencing), and analyzed using bioinformatic tools (e.g., genome assembly and taxonomic assignment) FAIR, comparable, and reproducible.

**Table 1:** Examples of metadata that support data management from sample to publication, and resources to help standardize data/ metadata and sharing (protocols, controlled vocabularies/ontologies, etc.).

Data management stage	Metadata fields	Standardize public resources
**Sample**	Latitude, longitude, date/time, temperature, biome/ecosystem, depth and/or elevation of sampling site, etc.	Environmental Ontology (ENVO), Minimum Information about x Sequence (MIxS), International Geo/General Sample Number (IGSN)
**Preparation**	Laboratory protocol(s): DNA extraction, purification, amplification.	Protocols.io, e-laboratory notebook/management software
**Data processing**	Software tools for QA/QC, assembly, annotation. Include reference (if published), version, and parameters used.	Community guidelines for describing and citing software [23–25]
**Feature**	E.g., Annotations of sequence data, such as taxonomy or function	NCBI Taxonomy, Genome Taxonomy Database toolkit (GTDB-tk); Gene Ontology (GO), Kyoto Encyclopedia of Genes and Genomes (KEGG), etc.
**FAIR: Findable (i.e., PID metadata)**	Data owner(s), organization, keywords	ORCID, Researcher Organization Registry (ROR); keyword selection [26] to enhance search engine optimization (SEO)
**FAIR: Accessible**	Usage license, privacy protocols, transfer protocols	Creative Commons, HTTP
**FAIR: Interoperable**	Type and size of data, file formats, etc.	.csv,.tsv, etc.
**FAIR: Reusable**	See data processing.	Workflow notebooks (e.g., [27])

In order for your data to be reused effectively and appropriately [20], we recommend providing as much metadata as possible.

### Rule 3: Data management plans are your first research product

Now that you have mastered the complexity (or at least scratched the surface) of what it takes to create FAIR, comparable, and reproducible data, we need to talk about data management plans (DMPs). These are often required by funders as supplementary documents to research grants, where you outline when, where, and how data from the project will be preserved and shared. We won’t go into best practices for creating a DMP, as that is well articulated by Michener [28]. However, we do want to emphasize that DMPs are no longer just supplementary pdfs. They can (and should) be created as FAIR, machine-actionable, living documents [29]. DMPs establish the initial node in your upcoming research product network (data, code, etc.). DMPs connect the people and data to the funding agency and put a stake in the ground for the work you are funded to complete. Check out DMPtool [30] to see if your funder already has an approved DMP template. Major funders like the NSF, NIH, and the US Department of Energy (DOE) already do. Your DMP DOI creates that first linkage between your research and your funding agency, which expands into a network that includes data, data creators and data maintainers, and eventually publications!

### Rule 4: Get your PIDs to work together

PIDs have a range of function and utility and can provide ways to connect and link a variety of research components, including physical samples, instruments, organizations, digital objects, and even individual people. PIDs also have metadata that use standardized relationship terms to capture how diverse research products and people are linked. What does all this enable? Automated generation of network graphs that connect you and your research products to the broader publishing landscape. Why is that useful? These links could illuminate connections that researchers might not have been aware of previously. And, perhaps most importantly, PIDs and their relationships enable individual researchers to be credited for research contributions at a granularity that was not previously possible. If you don’t have an ORCID iD (a PID for individuals), get one. In fact, since October 2019, NIH has required an ORCID iD for any research training, development, or education proposals. Other advantages? An ORCID iD enables you to move between jobs, organizations, and institutions without having to constantly update contact information. Many websites, including journal submission pages, now allow ORCID authentication, removing the need to change or update your institutional email login. ORCID iDs enable everyone—from students to research technicians to senior PIs—to have their contributions to scientific knowledge recognized and accessible to both peers and machines.

There are several other 10 Simple Rules that mention PIDs, including Contaxis [18], Hart [19], and Goodman [39]. Our main takeaway: Adopting PIDs and connecting them using standardized terms enables the publishing infrastructure to build your research network graph for you. Keep reading to find out why you should care.

### Rule 5: Share your data as openly as possible

The culture of data sharing is changing. The National Academies of Sciences, Engineering, and Medicine’s “Open Science by Design” [40] is an excellent report on where open science is headed. The current changes towards open science and data sharing are in part because of mandates from funders and publishers, but also because of support and incentives provided by societies and open science movements. In 2021, the American Society for Microbiology launched the ASM Data Prize [41], which “recognizes distinguished research achievements to support open data practices, development of standards and processes for data quality and sharing, and data workflows and management best practices that have advanced the microbial sciences.” The American Geophysical Union (AGU) has had a position statement on free and open science since 2011 and is one of the leaders in advocating for giving credit where credit is due [42–45]. And to top it off, NASA has launched a “Transform to Open Science” (TOPS; [46]) mission and declared 2023 as the year of open science, emphasizing the role open science has in broadening participation by historically excluded communities (see also [47]).

Now, with the connection of data PIDs, via their rich machine-readable metadata and standardized relationships, there are ways to enable open science *and* get credit for your data contributions. In 2011, Tenopir and collegues surveyed more than 1,400 researchers from disciplines ranging from environmental and computer science to medicine and reported that “the most important condition for sharing their [researcher] data was to receive proper citation credit when others use their data” [48]. A follow-up study several years later found that more researchers were sharing data but still with the caveat that they needed to publish the data first [49]. By assigning DOIs to data and connecting to PIDs such as ORCID iDs, the infrastructure is now in place for tracking and crediting individual data contributions in a transparent, automated way.

Want to make sure your peers know your data is available for reuse? Creative Commons provides several options for permitting reuse of digital resources, and many data providers and repositories either have default data policies (e.g., Dryad CC0 [50]) or enable you to select your preferred data policy/license upon submission (e.g., Zenodo). Consider applying CC0 to your data, which puts it in the public domain and removes all reuse restrictions. Another common option is CC-BY-4.0, a formal license that enables use without restriction but requires attribution. Unfortunately, it doesn’t appear to be clear when or how that might be enforced [51], and CC0 doesn’t remove the expectation that your data should be cited. Science has a culture of citing publications, and that same can and should apply to data. Regardless of your choices, be sure to read Rule 7 on how to make citing your data easy for others.

Finally, open science isn’t limited to sharing data. Other research products, such as software and models, can also be shared and cited. For cases when the research product is too large to be shared effectively, as is the case for some modeling outputs, there are guidelines that discuss what components and metadata can and should be preserved to support understanding and reuse. Additional guidelines at AGU [52].

As referenced in Rule 1, we acknowledge that not all research should be made fully open, out of respect for people and cultures that might be impacted. Be sure to adopt privacy protocols that are appropriate for your research (see Rule 8 in [19]), and acknowledge that data should respect the CARE (Collective benefit, Authority to control, Responsibility, and Ethics) Principles, especially with respect to indigenous data governance [53].

### Rule 6: Understand the roles of data generators and data repositories

Navigating the different data use policies across the research landscape is… complicated [54], but there is a general movement by funders and government agencies, including recommendations in 2013 by the US Office of Budget and Management [55] and Office of Science Technology and Policy (reviewed in 2016; [56]), to make publicly funded data public.

If your data are generated by an outside organization, ask if they support PIDs (Table 2). By assigning PIDs early and often in the data management lifecycle, your research components can be linked across the PID landscape (e.g., DMPTool, ORCID, DataCite) as they are generated instead of scrambling to meet data sharing requirements when you are ready to publish. Actually, we appreciate that publishing always requires some level of scrambling, but emphasize that your research components (data, software, etc.) could begin to collect usage counts and citations *before your publication is even submitted*.

**Table 2:** Common PIDs and possible relationships between them.

PID	Identifies…	Relationship between PIDs and more information
ORCID iD [31]	People doing the science	Example contribution roles via Contributor Roles Taxonomy [32]: data curation, formal analysis, funding acquisition, investigation, methodology
DOI	Digital objects: DMPs, data, software, publications, proposals, protocols	DataCite Schema [33] relationship types: cites/is cited by; supplements/is supplemental to; references/is referenced by; is funded by
ROR ID [34]	Research organization where science happens	See Research Organization Registry (ROR) to search for organizations
IGSN [35]	Physical samples collected and processed to generate data	See International GeoSample Number (IGSN); allows for parent–child relationship between samples; partnered with DataCite to track relationships [36]
RRID [37]	Resources (e.g., antibodies, model organisms, and software projects) used in the biomedical field	See Research Resource Identifiers (RRIDs); provides citation recommendations for use within publication text, often in the methods section
RAiD [38]	Collection of PIDs generated by a research project	See Research Activity Identifier (RAiD) for more details

If you are the data creator and responsible for releasing your own data, consider applying Rule 5 by submitting data to repositories that support PIDs and licenses that allow your data to be reused without restriction. *Be sure to also provide clear guidelines for citing your data*. The repository may already have guidelines, but if not, we recommend including a full data citation in the file metadata. See Rule 7 for a recommendation on formatting data citations.

Everyone consumes data, so it is critical that we all adopt best practices for citing data created by others in our community. The remainder of the rules outline how and why.

### Rule 7: Get that data citation

*If you are using data (yours or someone else’s) in a publication, cite it in the reference section of your publication. This is how data DOIs are automatically linked as “is cited by” (Table 2) to publication DOIs.* If the data creator or repository hasn’t clearly provided a data citation, send them a copy of this article and ask them for one! If that fails, or if you need to create a data citation for your own data, we recommend adopting the Earth Systems Information Partner’s (ESIP) data citation guidelines [57,58].

In brief, a data citation should include (paraphrased):

- **Creator/Author**: Person, people, or organization(s) responsible for the intellectual work to develop a data set. Consider including contribution roles as well. For example, CRediT (Contributor Roles Taxonomy) provides standard vocabulary for 14 roles: Conceptualization, Data curation, Formal analysis, Funding acquisition, Investigation, Methodology, Project administration, Resources, Software, Supervision, Validation, Visualization, Writing–original draft, or review and editing [32].
- **Public Release Date**: When the source data was first made available for use.
- **Title**: Formal title of the data set. It is recommended that version information be independent of the title. Note this is the title of the data set, not the project or a related publication. It is important for the data set to have an identity and title of its own.
- **Version ID**: Careful versioning and documentation of version changes are central to enabling accurate citation. Enables tracking as part of the citation for any version greater than 1.
- **Repository**: Name of the entity that holds, archives, publishes, prints, distributes, releases, issues, or produces the data. If citing processed data, this should also include a reference to the repository that holds the raw, source data.
- **Resolvable PID(s)**: Unique identifier that provides the ability to access the data. If citing processed data, this should also include a reference to the identifier for the raw, source data. Not all data have PIDs or can be digitally accessed, so an alternative method to access metadata, such as a URL or a physical address, can be provided instead.
- **Access Date**: Data can be dynamic and changeable in ways that are not always reflected in release dates and versions, so it is important to indicate when data were accessed.

If the data creator has not yet made their data available, it is still good practice to ask data creators for permission to use their data, especially if the data do not have a PID and guidelines for reuse. Then, encourage them to join the data citation movement with you!

Finally, and somewhat still underdevelopment, is how to cite data when you are working with large meta-analyses. We recommend contacting your preferred data repository early to ask about “Collection DOIs.” A collection DOI would be a new data DOI for your aggregation of public data, but that “cites” all of those data sets directly in the new DOI metadata. For example, at the DOE Systems Biology Knowledgebase (KBase), we enable users to access public data for comparative analyses and track provenance for each data object analyzed by a KBase user in our Narratives, which are built on Jupyter notebook [59]. When users are ready to publish their workflows, we work with the DOE Office of Scientific and Technical Information (OSTI) to issue DOIs that contain all data and reproducible analyses, including citation references to any public data contributed by others. We are still building out this functionality and welcome community feedback. More information is available at [60].

### Rule 8: Link research products to publications (and back again)

Publications will always be an important component of the scientific process. All research products (software, analysis notebooks, protocols, etc.) can be linked to publication DOIs, creating the final connections in your research product PID graph, which all started with and are connected to your funded DMP. In order for publishers to effectively make those connections, be sure your PIDs are released and contain accurate and comprehensive metadata and relationship types. Then, include those PIDs in *both* the publication data availability statement (if relevant) *and the reference section*. The reference section is the source of machine-readable, PID relationships that link across the publishing infrastructure. The data availability statement is currently *not* treated as a citation. Instead it provides human-readable context and quick access to the data.

One caveat—the current infrastructure does ***not*** effectively update the existing PIDs with the publication DOI once the article is released. In order to create that bidirectionality in the graph network, researchers still need to manually connect the PIDs by informing the repository of the data DOI or by updating the record directly. The data citation movement is working to streamline this process, but until then, we are grateful for your additional effort on this and your patience!

### Rule 9: Praise FAIR, comparable, and reproducible data

When reviewing proposals for funders or publications for journals, use these 10 simple rules on getting and giving data credit to promote broader adoption of the data citation movement. First, ask the funder or publisher if they have guidelines or a checklist for evaluating data. If they don’t, let them know you would appreciate that resource for future reviews. Either way, you can still evaluate if the authors made their data FAIR. Data and other research components should be referenced by a PID within the proposal or publication, and that PID should resolve to a location where the data are made available, ideally with clear data citation and reuse guidelines. It doesn’t take much time to confirm the PID is valid. If you want to explore further, try accessing the data and exploring to see if it is interoperable and easily reusable. If any step in FAIR is challenging, or the data are not available, please provide authors with constructive feedback. If the data are FAIR, please commend your fellow FAIR data advocates.

If you run a lab, consider employing a FAIR checklist as part of your student, postdoctoral, or technician departure protocol. Cornell University Research Data Management Service Group has a nice checklist to get you started [61]. Not only does it help reinforce data management best practices as part of their training, but it will greatly assist you in finding and reusing legacy data!

Finally, if you are reviewing candidates for hire or promotion/tenure, ask the committee if they are willing to consider preprints and open research products (like data and software) as criteria for evaluation. While it is still early days in the PID connection landscape, consider exploring the applicant’s ORCID record/profile or their DataCite Commons record. Many research products are not reliably available (yet) on either platform, but they are coming soon! These platforms enable researchers to display all their contributions, not just publications. And once the culture of citing data gains momentum (check out Rules 9 and 10 in [39] for encouragement), these profiles will provide information and links to all research products.

### Rule 10: Check your data metrics

Now that you know *how* to join the data citation movement, we acknowledge actually doing the data citation properly requires extra effort. Rule 10 explains *why this extra effort will be worth it*. In brief, it’ll help the publishing infrastructure help your career.

DataCite, the aggregator of PIDs for research products, has created the DataCite Data Commons [62] as a way for you to actually visualize reuse of your data (views, downloads, and citations). Data providers and repositories can join DataCite as members or DataCite Consortia and upload any reuse counts for sample and data PIDs they track. And, since it is important for these counts to be transparent and consistently reported, all reporting organizations must adhere to the COUNTER Code of Practice for Research Data [63].

In the not too distant future, you’ll have a funded DMP with a DOI that links to a series of research products (samples, software, e-lab notebooks, protocols, data, and journal articles). These products are shared widely with the community, and all reuse is automatically tracked, demonstrating the true reach of your research contributions. And while robust measures of impact as official metrics takes time, by building a solid infrastructure, and wide community adoption (described as part of the Make Data Count initiative [64]), you can begin to see how some effort now—to get and give credit for data (and other research products)—is worth it.

## Conclusions

By making data FAIR, with supporting information that also enables comparison and reproducibility, we begin to enable automated aggregation and meta-analysis of scientific products. As more complex questions are turning to machine learning and artificial intelligence as tools, well-documented, FAIR data and good management are critical. As is ensuring that everyone who has contributed to the advancement of scientific knowledge receives attribution. Effective, efficient, and transparent credit for sharing of data and other nonpublication research products has the potential to greatly accelerate the growth of high-quality, reproducible, and transferable knowledge [20].

Don’t have a data project or publication in the works at the moment? We recommend getting your PIDs linked up anyway! Check out AGU’s “Your Digital Presence” [65] for a few steps everyone can do today.

Are you now a data citation expert now and ready for the next challenge? Check out Contaxis and colleagues’ [18] 10 simple rules for improving research data discovery. Their article covers many of the same topics but also explores data schemas and ontologies as the next step in data management skills. Terms we hint at, as they make up names of recommended resources (e.g., the Environment Ontology), but do not explore in depth. There is a lot to explore, for those interested in joining us in making data citations work for everyone.
